# Foraging and Food Selection in a Desert Rodent: Diet Shifts of the Sandy Inland Mouse between Population Booms and Busts

**DOI:** 10.3390/ani13101702

**Published:** 2023-05-20

**Authors:** Stephanie J. S. Yip, Christopher R. Dickman

**Affiliations:** Desert Ecology Research Group, School of Life and Environmental Sciences, The University of Sydney, Sydney, NSW 2006, Australia; chris.dickman@sydney.edu.au

**Keywords:** arid zone, Australia, desert rodent, diet, foraging, granivory, invertebrates, *Pseudomys hermannsburgensis*, seeds

## Abstract

**Simple Summary:**

Rodents in Australia’s central deserts face highly unpredictable climatic conditions, with long dry ‘bust’ periods, when resources are sparse, punctuated by brief ‘boom’ periods, after heavy rainfall, when resources are abundant. We studied the diet of an Australian desert-dwelling rodent species, the sandy inland mouse *Pseudomys hermannsburgensis*, by watching what animals ate in the field and by analysing the stomach contents of preserved specimens collected opportunistically over 24 years. Both techniques showed that seeds were the most important component of the diet, and that invertebrates and green plant material were also consumed. Although there were no seasonal or sex-based differences in diet, invertebrates were consumed more frequently during bust periods compared to booms, perhaps suggesting that animals switch to invertebrates at times when seeds are scarce. We drew two main conclusions. Firstly, sandy inland mice are omnivorous. This contrasts with a common view that seeds generally are the mainstay of desert rodents but supports some previous research on Australian desert species. Secondly, in environments with unpredictable climatic conditions where food resources are likely to be unreliable, dietary flexibility is important in allowing animals to exploit different food groups as these become available at different times.

**Abstract:**

Seeds are commonly viewed as the mainstay of the diet of desert rodents. We describe the diet of a common Australian desert rodent, the sandy inland mouse *Pseudomys hermannsburgensis*, using direct observations of free-living animals and analysis of the stomach contents of preserved specimens. Direct observations showed that animals forage mostly on the ground surface and eat seeds from a wide range of plant species, as well as invertebrates and occasional green plant material. Stomach content analysis revealed no differences in the presence or absence of these three major food groups between seasons or the sexes. However, invertebrates were more prominent in the diet of mice during prolonged, dry, population ‘bust’ periods compared with post-rain population ‘boom’ periods, with this dietary shift probably reflecting a scarcity of seeds during the busts. The results confirm that seed is an important component of the diet of *P. hermannsburgensis*, with 92% of stomachs containing seed. The results also support the classification of the species as omnivorous rather than granivorous, with 70% of stomachs containing invertebrates and over half the specimens analysed containing both seeds and invertebrates. We suggest that dietary flexibility is important for rodent persistence in Australia’s climatically unpredictable arid regions.

## 1. Introduction

Compared to desert-dwelling rodent species in many parts of the world (North America, South America and South Africa), where dietary studies have often been a focus, there is a lack of detailed information relating to the foods selected and eaten by native rodents in Australia’s extensive arid regions [1,2,3,4]. In North America, for example, heteromyid rodents have been much studied, and the primarily granivorous diets and seed-caching behaviours of many species set early expectations that all desert rodents could be expected to be granivorous, e.g., [5,6,7]. In South America, seeds comprise a large proportion of the diets of many species, notably in arid regions, but invertebrates, fungi and green plant material often form additional components of the diet [8,9]. In Africa, the consumption of seeds and green plants by rodents has stimulated much study in many cropping areas due to the destructive impacts of species such as multimammate rats (*Mastomys* spp.) [10,11]; however, desert rodents have also been subject to considerable study, with some authors suggesting that granivory is the norm for small and medium-sized species [12]. Relatively fewer rodent species occur in Australia than in other continental regions, but quantitative analyses of diet are still limited and distributed patchily among extant taxa, e.g., [13,14].

The sandy inland mouse, *Pseudomys hermannsburgensis*, provides a good example of a native species for which only limited dietary information is available, with most of the detailed research conducted more than 20 years ago, e.g., [14,15,16,17,18]. The relative paucity of relevant information is surprising in that the sandy inland mouse has the largest extant distribution of any Australian native rodent and is often the numerically dominant member of desert mammal assemblages [19,20]. This paper focuses on the foraging behaviour and diet of *P. hermannsburgensis* in sand dune habitats in central Australia.

Foraging in *P. hermannsburgensis* usually occurs in or near microhabitats that provide dense cover which, in sand dune habitats, is mainly amongst hummock grasses on the sides of dunes and in the dune swales [17,18]. This species is nocturnal and quadrupedal with an average mass of 12 g [17]. In early work it was believed that the species was almost solely granivorous, this classification being based on the diets of ecologically similar species that had been studied in deserts in other parts of the world [7,12], and on the examination of small samples of four to six sandy inland mouse stomachs [21,22,23]. Murray and Dickman [15] and Murray et al. [14] later suggested that at least 10 individuals are required to reliably characterize the dietary diversity of small desert rodents. More detailed analysis of the diet of *P. hermannsburgensis* with larger sample sizes revealed the species to be omnivorous and to have a variable diet containing invertebrates, seeds and plant material [14,15,16]. While *P. hermannsburgensis* may be best classified as a generalist or an omnivore, seeds are still a major component of its diet, with the proportion and relative importance of seeds differing over time and potentially over different seasons [15] and at different times in the erratic boom-and-bust cycles that characterize the Australian desert environment [24,25]. Rainfall in central Australia is typically heaviest in spring and summer, driving pulses of seed production that then diminish with the onset of cooler and drier conditions in autumn and winter [14,17,24]. Very heavy rainfall events in spring and summer (e.g., >90th percentile) generate much larger pulses of primary productivity and seed production and often stimulate population booms in desert rodents, whereas years with drier summers lead to bust conditions of resource exhaustion when populations of desert rodents are small [17,24].

This study used two complementary methods to describe the diet of *P. hermannsburgensis*: direct observations in the field and stomach analysis. Direct observations can provide detailed insight into the foraging mode of the species being observed as well as document food items that are selected or rejected. However, as *P. hermannsburgensis* is nocturnal and cryptic, making observations can be time-consuming and fraught with challenges to ensure that normal foraging is not disturbed. By contrast, faecal or stomach analysis provides a snapshot look at the foods consumed during one bout of foraging but can be challenging, because food items are often finely comminuted and partially digested, making identification difficult. In this study, stomach analysis was carried out, rather than faecal analysis, for a number of reasons. Firstly, stomachs from various seasons were readily available as these had been collected during previous long-term research by the Desert Ecology Lab at the University of Sydney; secondly, there are some advantages in using stomach contents versus faeces, or scats, such as the food items being less digested. This makes food items easier to identify and reduces the time spent on analysis [26]; further, scats often return less reliable results than stomach contents, especially for seed-eating rodents, due to the greater degree of digestion of material that has passed the length of the digestive tract [27]. Based on previous observations, we expected seed to be the predominant food type in the diet of *P. hermannsburgensis*, but also that the relative importance of seeds and other food types would vary with calendar season and with boom-and-bust conditions. Specifically, we predicted that seed consumption would be greater during (1) spring and summer than during autumn and winter, and (2) population boom compared with population bust periods. In view of the ability of the study species to consume a diverse range of food types, we expected that a decline in seed in the diet would correspond with an increase in invertebrate and/or green plant material. 

## 2. Materials and Methods

### 2.1. Study Site

Research was conducted on Ethabuka Reserve (formerly Ethabuka Station) in the north-eastern Simpson Desert, western Queensland, Australia (23°46′ S, 138°28′ E). The landscape has long parallel sand dunes up to 8 m high and 0.5–1 km apart, with hard claypans forming between the dunes [15]. The major vegetation is spinifex *Triodia basedowii,* with ephemeral herbs and perennial shrubs, such as *Crotalaria* spp. and *Grevillea* spp., dominating the dune crests; in the swales there are stands of trees such as *Eucalyptus* spp., mulga *Acacia aneura* and gidgee *Acacia georginae* [28].

The average rainfall can vary greatly, oscillating between periods that are either dry and unproductive (‘bust’ periods) or wet/flooded due to extreme rainfall events that cause brief pulses of high productivity (‘boom’ periods). Most rainfall occurs in summer, with occasional heavy falls locally and regionally at other times [29,30]. Over an average of 94 years, 199 mm/year of rain was measured at Marion Downs (a station located 120 km from Ethabuka), but rainfall can differ significantly between years. For example, an average 214.2 mm fell in 1999, and a well above average 496.6 mm of rain fell in 2000 [29,30]. Unlike in hyper-arid deserts, some rain falls every year. Temperature varies widely depending on season, with the average daily maximum temperature exceeding 40 °C in summer; during winter temperatures often fall below 5 °C [30]. Following Murray and Dickman [15], summer was taken to cover the months of December to February, autumn from March to May, winter from June to August, and spring September to November. Over the course of this study, significant rainfall events (>90th percentile) occurred over the spring and summers of 1990–1991, 2000–2001, 2010–2011 and 2015–2016, resulting in population eruptions of *P. hermannsburgensis* and many other consumer species [31,32,33,34,35,36]. 

### 2.2. Direct Observations of Foraging

Sandy inland mice were observed in the field on 25 visits to the study site between April 2008 and June 2022. Most of these animals had been captured in pitfall traps set in the sand dune environment on the previous night, using trapping protocols that have been described in detail in Dickman et al. [32,33,37]. The animals were removed from the traps and placed individually in perspex holding cages (24 × 16 × 20 cm high) that had been provisioned with a 1 cm substrate of sand, dry leaf litter and shelter (halved egg cartons) and a slice of apple to provide food and moisture. The holding cages were placed on-site in a cool, shaded position away from disturbance and left throughout the day. About 2 h after dusk when night had fallen, animals were transported in their holding cages and then released within 10 m of the pitfall trap sites where they had been captured. 

In early observations (2008–2015), animals (*n* = 32) were dusted with fluorescent pigments (Fiesta Daylight Pigments; Swada Ltd., London, UK) and the pigment trails followed using a UV blacklight after release to facilitate detection of animals’ foraging paths [38]. In other early trials, spool-and-line tracking was used [39]. Here, a spool of fine 2-ply cotton thread that unwinds from the inside (cocoon bobbins, Coats Australia Pty Ltd., Sydney, NSW, Australia) was glued to the nape of a sandy inland mouse using cyanoacrylic glue (“superglue”), and the free end of the spool tied to a shrub prior to the animal being released. The spools, weighing <5% of an animal’s body mass, were shed after the animal had moved away a distance of 80–100 m and the entire length of the spool had paid out. Animals (*n* = 37) were followed several minutes after they had been dusted and released with fluorescent pigment, or released with the spool, to provide some time for them to move into cover and resume ‘normal’ behaviour. The trail left by the fluorescent pigment or the spool line was followed quietly on foot to enable the investigator to find the animal without causing undue auditory disturbance. In all situations, a red torch (hand-held Dolphin Energizer or Ledlenser H7R head torch) was used to locate animals and minimize visual disturbance. This approach allowed the observer to approach to within 2–3 m of focal animals without causing any apparent change in behaviour, although recent work does suggest that rodents have some capacity to detect red light illumination [40]. 

In later observations of sandy inland mice (2016–2019, *n* = 11), animals were captured and maintained over the course of a day as noted above, but were provided with a small cyalume fishing lure (4.5 mm × 29 mm; Nightlight, Aerostar Ltd., Sydney, NSW, Australia) that was glued to an animal’s nape using cyanoacrylic superglue. These lights emanate a weak green glow that lasts for 3–4 h before fading; pilot trials showed that the lure was usually shed by animals overnight or over the course of the next day. Although animals were still followed using red torchlight after their release near their point of capture, the weak green light was easier to find in the dark. Animals were likely able to detect these lights, but there was no evident effect on their behaviour when moving around or foraging. 

In addition to using fluorescent pigments, spools-and-lines and cyalume lights, animals were sometimes (*n* = 8) detected during the course of the observer moving about on the dunes at night, and these animals were followed and observed when opportunities arose. Once detected, animals were followed using red torchlight, and any foraging events were observed. There were no differences in the behaviour of animals or in the duration of time they were observed using the different techniques. It was usually possible to see when animals had stopped and picked up a food item, except when animals had moved under dense cover, and it was often possible to identify what the food item was. Seeds could often be identified to species, with identification aided by the use of close-focus binoculars. After animals had moved on from a foraging event, the site was inspected to confirm the identity of food items; sometimes, parts of seed husks could be found, or intact seeds that had remained buried or otherwise not exploited by the forager provided confirmation. If animals had dug into the soil, the extent and depth of the excavation was measured using a ruler. Green plants could usually be identified readily when animals stopped to feed on them, with confirmation made after the forager had moved on. However, invertebrates could be identified only at the time of capture, and the level of identification was usually coarse (e.g., spider, beetle). Descriptive notes rather than formal ethograms were made for all foraging events, and times spent following animals were recorded. All observations were made by the authors to ensure consistency in recording. 

### 2.3. Collection of Stomach Material and Diet Analysis

Specimens were collected over a period of 24 years on research trips to the Simpson Desert. Some individuals had died in pitfall traps, others were collected during specific research projects, e.g., [17,18,24,41,42], and others were collected opportunistically by landowners on Ethabuka and neighbouring properties to the immediate north of Ethabuka. All specimens were initially preserved in 10% formalin, then placed into 70% ethanol in specimen jars. There was a total of 186 sandy inland mouse stomach-content samples. In total, 8 specimens had no identification tags for date or location collected, and the stomachs of 2 specimens were empty, leaving 176 fully labelled and provenanced stomach-content samples from the study site. These were collected during different seasons and boom-and-bust periods between 1991 and 2014, and comprised 102 female, 72 male and 2 unsexed individuals.

Previous research [15] determined the minimum number of stomachs required to reliably determine dietary diversity of each seasonal population sample for *P. hermannsburgensis* to be 10 individuals. This minimum number was obtained by plotting the cumulative number of stomachs against the cumulative number of food categories that could be recognized in the diet, with the graph levelling off at *n* = 10 samples. The stomachs of the mice were removed and the contents analysed; the contents of the intestines were not examined as these contents were usually too digested to allow reliable identification of food types. 

Individual stomach contents were initially photographed using a Leica M205 C microscope; the contents were first washed with water to remove extraneous material such as hair or grit and then washed through a 125 µm sieve to remove particles too small to identify [14,15] Then, the contents were spread out on microscope slides and inspected in detail. Contents and food fragments were photographed using the same microscope as previously and scored for presence or absence on a per-stomach basis.

Contents were initially separated into three major food classes of seed, non-seed plant material and invertebrate, with only the seed group identified to species. No vertebrate or fungal material was detected in any samples. A reference collection of seeds collected previously at the study site was used to identify the seeds, with external characteristics such as seed coat used to determine seed species. 

### 2.4. Statistical Analyses

The direct observations of foraging were largely descriptive but allowed tallies of three major food types (seeds, invertebrates and green plants) to be carried out. Observations made using all three tracking methods were combined to provide an overall tally and to ensure that sufficient observations were available for analysis. A chi-squared goodness-of-fit test was used to determine whether the proportional frequency of items differed between these three major food categories. To test whether the representation of these food types differed by season or boom versus bust conditions, chi-squared contingency tests were employed. 

To test whether the dietary composition of the stomach samples differed by season or boom versus bust conditions, chi-squared tests were again used. Because sample sizes were larger for stomachs than for the direct observations, further analyses were carried out to compare seasonal and boom-and-bust diets, with years as replicates. For this, we carried out permutational multivariate analysis of variance (PERMANOVA), with 999 permutations, implemented in the ‘vegan’ package in R version 4.2.2 (https://cran.r-project.org/bin/windows/base/) [43]. Sex was included as an additional factor. Statistical significance was accepted for any factors associated with a *p*-value ≤ 0.05. If significant results were obtained, we used the similarity percentages (SIMPER) procedure to detect where differences occurred, again using the ‘vegan’ package.

## 3. Results

### 3.1. Direct Observations

Over the course of 25 field visits, 88 sandy inland mice were followed and observed for a total of 163 h. A total of 23 individuals moved quickly to sites under cover after release and stayed immobile for periods of at least 60–90 min, after which observations were terminated; 65 individuals were followed and observed for periods of 5–172 min (116 h total) until they moved out of sight or observations were terminated. Of these 65 individuals, 53 were observed to consume food items; 38 individuals were observed to eat a single food item, and 15 stopped to eat between 2 and 4 food items, yielding a total number of 82 foraging observations when the main food type could be identified. There were at least 13 further occasions when animals appeared to stop and eat, but this could either be not confirmed or the food item not identified due to the orientation of the animal or obstructions that precluded a clear view; these incidences were excluded from further consideration.

When foraging, animals either moved slowly and apparently purposefully with the head close to the ground surface, or in a stop-and-start mode where they moved quickly from one site to another, usually less than 1 m apart, before slowing down and investigating the new site. Investigation took the form of sniffing at the ground and superficial digging, usually to a depth of no more than 1 cm, but on 2 occasions, to a depth of 2.5 cm, with the animal often remaining within a focal area of no more than 25 cm × 25 cm until moving to a new site. In 67 of the 82 foraging observations, animals held the food item in their forepaws and ate it at the site where the item had been found; in 15 observations, the food item was moved to a nearby site that provided more cover than that where the item had been found. Food items were carried in the mouth. Of the 116 h that animals were in view, only 69 min were spent eating (mean ± SD: 50.49 ± 14.30 s per food item). The sandy inland mice appeared to be very vigilant for much of the rest of the time they were observed, either sitting immobile under cover, moving with ears erect, or pausing with the head up and one forepaw on the ground in an ‘indecision-alert’ posture. Animals were easily startled if the observer made a noise or if other minor disturbances were perceived, and either moved quickly under cover and remained immobile or adopted a vigilant stance until movement was resumed, usually several minutes later.

Of the food items that animals could be identified as consuming, 65 were seeds, 11 were invertebrates and 6 were leaves, stems or other green plant parts (χ^2^ = 78.33, 2 df, *p* < 0.001). There was no association between the frequency of food types eaten with either season (χ^2^ = 7.84, 6 df, *p* = 0.25) or boom-and-bust conditions (χ^2^ = 0.23, 2 df, *p* = 0.88) (Table 1). Because these tests included data from 15 individuals that had eaten 2–4 food items, hence violating the assumption of independence, we randomly selected only 1 of the food items eaten by these 15 individuals and repeated the tests with a total *n* = 53. The results were very similar to those when all observations were included. 

Seeds that could be confidently identified as being consumed by sandy inland mice included the grasses *T. basedowii* (*n* = 8) and *Aristida contorta* (*n* = 1); herbs and forbs *Goodenia cycloptera* (*n* = 3), *Haloragis gossei* (*n* = 1), *Trachymene glaucifolia* (*n* = 4), *Dicrastylis costelloi* (*n* = 2), *Newcastelia spodiotricha* (*n* = 2), *Trianthema pilosa* (*n* = 2), *Trichodesma zeylanicum* (*n* = 1), *Sclerolaena diacantha* (*n* = 1), *Sida fibulifera* (*n* = 1) and *Crotalaria* sp. (*n* = 1); and shrubs *Grevillea stenobotrya* (*n* = 11), *Acacia ligulata* (*n* = 3), *A. dictyophleba* (*n* = 2) and *Dodonaea viscosa* (*n* = 1). Although it was not quantified, animals whose fluorescent pigment trails were followed often passed seeds on the soil surface during their foraging explorations without stopping to investigate or consume them. These included seeds of *Crotalaria* spp., *Eremophila* spp., *Eucalyptus* spp., *Senna pleurocarpa* and *Stylobasium spathulatum*, all of which are large and conspicuous, as well as patches containing many smaller seeds of species such as *Portulaca intraterranea* and *Euphorbia drummondii*. 

Invertebrates that were eaten by sandy inland mice included beetles (*n* = 2), lepidoptera (*n* = 1), spiders (*n* = 3) and an unidentified insect larva. Green plant material included the succulent leaves of *Calandrinia balonensis* (*n* = 2) and *Portulaca intraterranea* (*n* = 1), and the stems of small herbaceous plants *Trachymene glaucifolia* (*n* = 1), *Oldenlandia pterospora* (*n* = 1) and fan flower *Scaevola depauperata* (*n* = 1). 

### 3.2. Stomach Content Analysis

**Figure 1:**
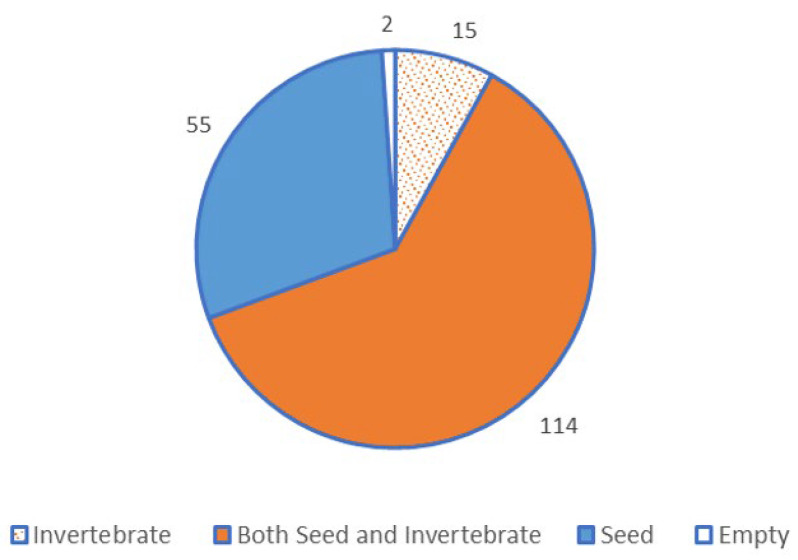
Frequencies of stomachs of sandy inland mice *Pseudomys hermannsburgensis* containing seeds alone, invertebrates alone and both food types together (*n* = 186), from specimens collected in the Simpson Desert.

Seeds, invertebrates and green plant material were the main food categories recorded in the stomachs of sandy inland mice, with seeds again predominating. Of the 176 stomachs that contained food material, 162 contained seed, 125 contained invertebrates and 39 contained green plant material (χ^2^ = 73.29, 2 df, *p* < 0.001). If the stomach samples from the 8 unprovenanced specimens are included, 169 contained seed, 129 contained invertebrate and 46 contained green plant material (χ^2^ = 68.66, 2 df, *p* < 0.001). Seed was, therefore, the major component of sandy inland mouse diet, with 92% of 184 stomachs (excluding the 2 empty stomachs) containing seeds, 70% containing invertebrates and 25% containing plant material. Overall, 62% of stomachs contained both seeds and invertebrates (Figure 1). Plant material was never the sole food type in any stomach, and it always comprised less than 10% of the stomach contents. 

In total, 6 seed species were identified, with 12 unknown species. The majority of the seeds identified were the grasses *Triodia basedowii*, which was identified in 81% of stomachs containing seed, and *Yakirra australiensis*, with dicotyledenous seeds of *Grevillea stenobotrya*, *Acacia dictyophleba*, *Trachymene glaucifolia* and *Ptilotus polystachyus* also identified. Green plant material could not be identified to species but included leaf, stem and root tissues, whereas invertebrates comprised insects and spiders; other invertebrate types were probably also present but could not be identified reliably. 

**Figure 2:**
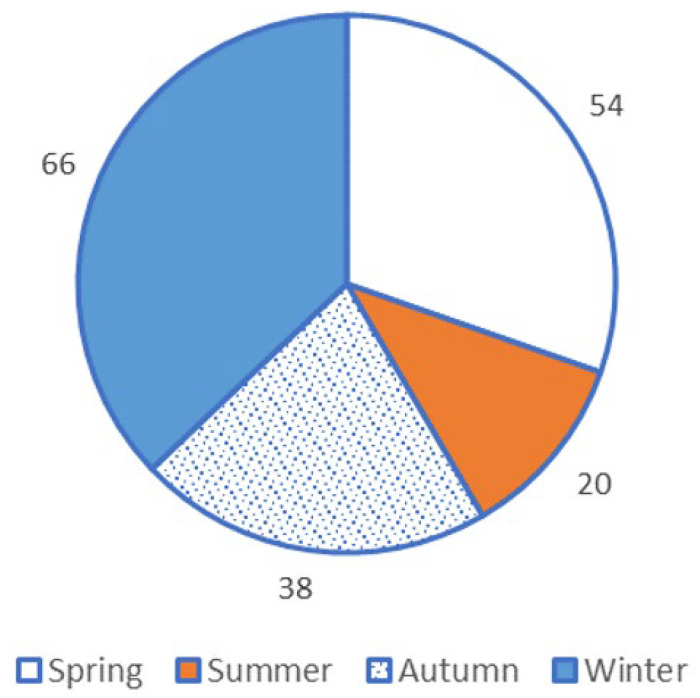
Number of stomach samples of sandy inland mice *Pseudomys hermannsburgensis* available for diet analysis, by season (*n* = 178, including 2 empty stomachs), from specimens collected in the Simpson Desert.

More stomach-content samples were available from winter than from the other seasons (Figure 2), but the distribution of food types eaten by sandy inland mice did not differ between the seasons (χ^2^ = 3.24, 6 df, *p* = 0.78) or boom-and-bust conditions (χ^2^ = 5.94, 2 df, *p* = 0.051), although there was a strong trend for association in the latter test due to an increase in representation of invertebrates in the diet during busts (Table 2).

**Figure 3:**
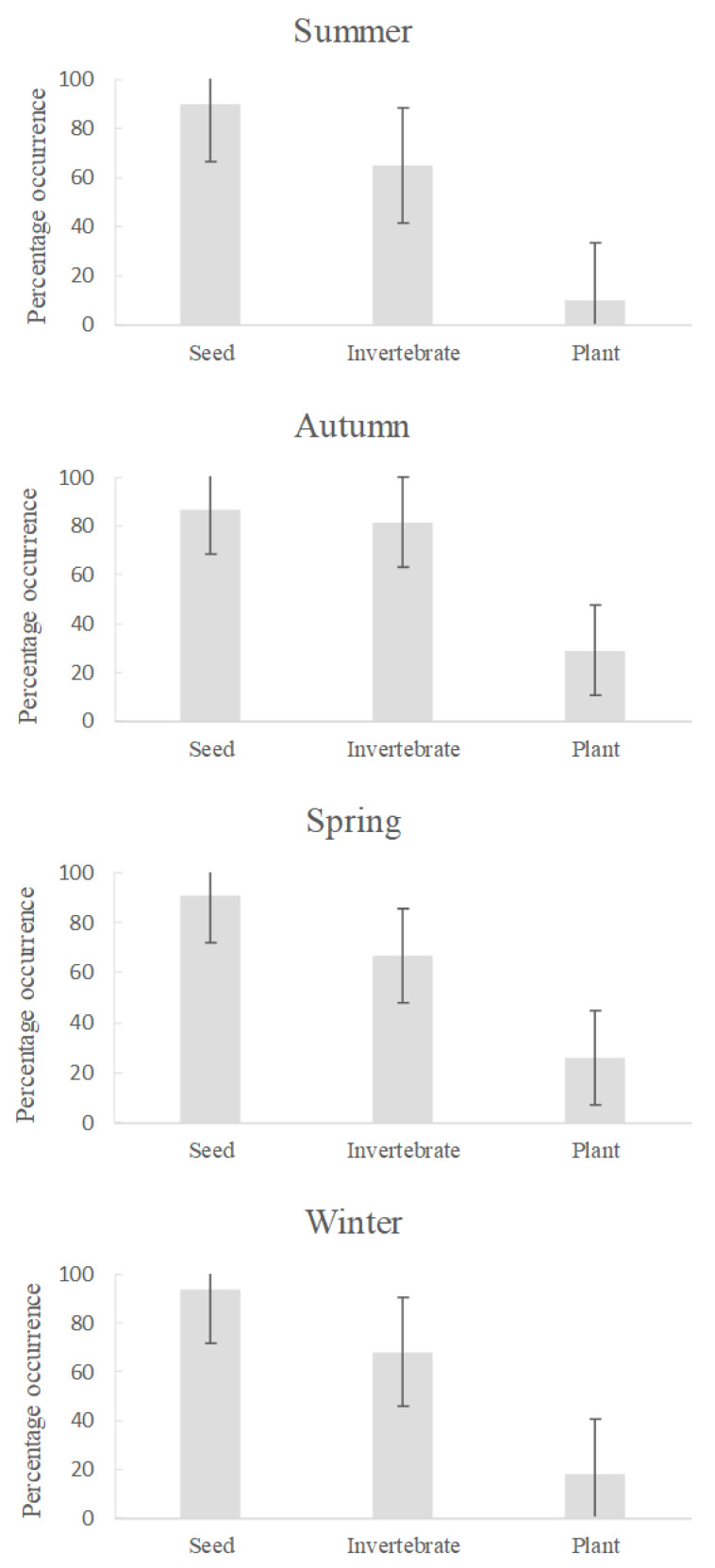
Percentage seasonal occurrence (mean ± SD) of each of the three broad categories of food in the diet of sandy inland mice *Pseudomys hermannsburgensis* from 176 specimens collected in the Simpson Desert. (Note that error bars are symmetrical about the mean values, but for clarity are truncated where they would lie below 0% or exceed 100%.)

The percentage occurrence of seeds in the diet was consistently high when data were averaged across seasons and years. Invertebrates also were present in the diet in all seasons, with a particularly high frequency of occurrence (80%) of invertebrate material during autumn (Figure 3). Green plant material was represented at low frequency in all seasons, with a small increase in autumn. There was, however, considerable variation between years in the representation of each food type in the diet (Figure 3).

In further analysis, PERMANOVA showed that there was no significant variation in the diet of sandy inland mice by season (df = 3, *p* = 0.304), nor by sex (df = 1, *p* = 0.872), and there was no significant interaction between season and sex (df = 3, *p* = 0.566). There was also no significant variation in diet between boom-and-bust periods (df = 1, *p* = 0.079). However, as the boom-and-bust comparison was close to being significant, a SIMPER analysis was run. This showed that there were significantly more invertebrates in the diet of *P. hermannsburgensis* during bust periods than during booms (boom average = 0.145, bust average = 0.583, SD = 0.164; *p* = 0.041). 

## 4. Discussion

Seed has been acknowledged as an important food resource for many desert-dwelling vertebrate and invertebrate species [44,45], especially rodents [46,47]. While invertebrates can be more beneficial than seed to consumers due to their relatively higher energy content, seeds provide other benefits. They require less energy expenditure to process, as seeds are immobile and, thus, require less energy to acquire and consume; different seeds likely contain different nutrients that may not be found in invertebrates; and seeds are often buried, creating seed banks that are an easy and consistent resource to be exploited by rodents [47]. Predavec [48] and Beh [41] were able to show the importance of the seed resource for a population of *P. hermannsburgensis*; while supplementary provision of seed did not reverse a population decline, extra seed slowed the overall rate of decline. There is, in addition, a linked relationship between seeds and desert rodents. While rodents rely on seeds for consumption and, ultimately, for their survival, there is some evidence that the fate of seeds, the seedbank and plant communities can be impacted by granivorous rodents [49]. The results in this study confirm that seeds are an important component of the diet of sandy inland mice, with a substantial number of stomachs containing seeds. This result supports previous research by Murray and Dickman [15], who also concluded that seeds are a major component of the diet of *P. hermannsburgensis*, with over 50% of stomachs containing more than 70% seed.

Despite the importance of seeds in the diet of the study species, animals also consumed invertebrates very frequently and green plant material less frequently. Flexibility in switching between the main food groups was less evident than initially expected, with no seasonal change in diet detected, but there was stronger evidence that invertebrates were taken at higher frequency in bust compared to boom periods, as we had predicted. This shift most likely reflects food resources, specifically seeds, being scarce during bust periods, as shown by Predavec [17] and Ricci [24], so the sandy inland mice supplemented their diet with invertebrates. Although rainfall can positively affect some invertebrate groups, such as ants [50], populations of many taxa respond negatively to rainfall, or appear unaffected by it and are thus relatively more available during dry bust periods than during booms [51]. A shift towards increased importance of invertebrates in the diet has been observed previously by Murray and Dickman [15], except that these authors saw this shift during autumn; their assumption was that dietary shifts reflected the relative availability of different food resource groups and, thus, that the increase of invertebrates in autumn diets could indicate increased availability of invertebrates and/or a decline in seeds. We also saw an apparent increase of invertebrates in the diet of *P. hermannsburgensis* in autumn (Figure 3), but the significance of invertebrates only became apparent when stomachs were separated by boom-and-bust periods. The suggestion that temporal shifts in diet reflect the relative availability of different foods [15] seems appropriate but awaits further confirmation by studies that simultaneously monitor food availability and the foods that are eaten.

Three further alternative, or additional possibilities, can be invoked to explain the increased consumption of invertebrates during bust times. First, foragers may search more actively for invertebrates during busts because they provide relatively greater amounts of protein, fat and minerals, such as calcium and iron, than seeds [16,52]. These components of food are likely to be more important to foragers during busts than booms because they take longer to digest and, thus, may reduce the risk of starvation when food is scarce. Dietary shifts can occur quickly. For example, at high latitudes when days are long (18 h daylight) in summer, bank voles *Myodes glareolus* switch from selecting high carbohydrate foods at sunset to foods with higher protein and fat content near sunrise to prepare for the day-long fast [53]. Second, during bust periods, sandy inland mice retreat to small and spatially isolated patches of woodland located between the sand dunes and remain confined there until the arrival of drought-breaking rains [37]. If the woodland patches contain relatively more invertebrates than the sand dune habitats, the diet shift in *P. hermannsburgensis* may simply reflect animals’ greater access to this food type in the patches. Alternatively, with the localization of the population in woodland patches, increased competition between mice for food is likely to rapidly deplete seed resources and, hence, prompt the diversification of the diet to include more invertebrates. Food resources and the diet of *P. hermannsburgensis* would need to be sampled at fine spatial and temporal scales to test these possibilities. Third, bust periods are characterised by dry conditions; succulent plant material is scarce and woodland patches contain primarily woody perennial shrubs and trees [28]. Under these conditions, animals may then consume more invertebrates to meet their requirements for water. However, this last possibility is perhaps the least likely: provision of free water had no effect on the consumption of dry food by *P. hermannsburgensis* in early experimental trials at the study site [54], and seed moisture content has little or no effect on either seed detection or consumption by the study species [55].

Our results confirm that *P. hermannsburgensis* consumes a broad range of food types, with the relative contributions of these foods changing over the boom-and-bust cycle. This could reflect dietary generalism, whereby animals eat different food types in correspondence with their availability in the environment, but simultaneous sampling of foragers’ diets and food resources is required in order to be certain. However, our results do confirm omnivory. The broad definition of an omnivore is that it is an animal that eats multiple different types of food or, more specifically, that the animal ‘shows no distinct, continuous preference for one particular food type, instead exhibiting a reliance on a number of different food sources’ [14]. This study, therefore, supports the classification of the sandy inland mouse as being omnivorous rather than granivorous, as proposed originally by Murray and Dickman [15,16], with 13% of direct observations showing animals eating invertebrates and over half the stomachs analysed containing both seed and invertebrate material. At least 11 of 16 desert-dwelling rodent species are confirmed or suspected to be omnivores in Australia; there may be more, but the diets of some species, such as the western pebble-mouse *Pseudomys chapmani*, are currently understudied or unknown [14]. Being omnivorous would be advantageous to Australian desert rodents, as rainfall is highly unpredictable and this, in turn, affects the reliability with which certain resources, such as seeds, will be available. It is notable that no Australian desert rodents are known to cache seeds for later use [20,56], in stark contrast to heteromyid rodents in North America and cricetid and sciurid rodents in other parts of the world [57]. This, presumably, reflects uncertainty that a seed cache, even a large one, may be able to sustain Australian desert rodents through long bust periods. However, by exploiting invertebrates and green plant material, rodents likely increase their chance of survival and decrease the risk of starvation compared to their prospects if they were limited to only seeds in their harsh and unpredictable environment [58,59].

The present study had a number of strengths and weaknesses. On the one hand, diets could be studied over a long time period owing to the availability of specimens that had been collected across boom-and-bust periods and from all 4 seasons over 24 years. Early studies [21,22,23] classified *P. hermannsburgensis* as granivorous but used small sample sizes of only four to six animals. Subsequent work [15] showed that larger sample sizes were needed to reliably classify the species’ diet, and concluded that if samples were collected only in winter or summer it could be assumed—erroneously—that the species is primarily granivorous. In this study, regardless of season (Figure 3), invertebrates appeared to play an important role in the diet of sandy inland mice, with sample sizes of 20–66 between different seasons showing substantial contributions of invertebrates to the diet at these times. 

On the other hand, this study had some key limitations. In the first instance, only a gross dietary analysis could be carried out on stomach content samples of *P. hermannsburgensis*, with only the presence or absence of the three major food classes of seed, invertebrate and plant material (non-seed) recorded. Most of the specimens were old, and the poor state of preservation of much of the stomach material meant that the most reliable level of identification was at the coarse food-group level. Food groups such as fungi were potentially present [60,61], but could not be determined. We did attempt to identify seeds to as fine a level as possible where these had been preserved well, but neither invertebrates nor green plant tissues were identified further. It would be advantageous to conduct more detailed dietary analyses by scoring the relative abundance of each specific food item by estimating its percentage occurrence in fresher or better preserved samples [15], to explore the utility of DNA-based mini- or meta-barcoding techniques to identify the full range of dietary items, e.g., [62], or to run cafeteria-style trials in the field or laboratory [63]. Except for cafeteria trials, these latter techniques have the advantage that they can be used on faecal samples. Secondly, this study lacked spatial variability, as all specimens were collected on or near Ethabuka, in the north-eastern Simpson Desert. *Pseudomys hermannsburgensis* is found across Australia in semi-arid and arid areas, often in hummock grasslands, but also in other habitats such as mallee shrublands and acacia woodlands [17,19,20,64]. There could be potentially much greater variation in the diet of sandy inland mice from different regional areas. For example, while a flexible diet is selectively advantageous for survival in unpredictable desert conditions [59,65], in semi-arid areas or areas where rainfall is more frequent and predictable, *P. hermannsburgensis* could be potentially more granivorous or herbivorous.

Interestingly, 38 specimens had worms in their stomachs. There has been extremely limited research on parasites in Australian desert rodents [66], and the stomach worms could not be further identified here. It is unclear whether these worms may have affected the health or behaviour of the infected animals, and studies of the prevalence and incidence of species of endoparasites remain to be conducted in both *P. hermannsburgensis* and other species of Australian desert rodents. 

## 5. Conclusions

The findings of this study confirm that seeds play an important role in the diet of sandy inland mice at all times of the year, and during boom-and-bust periods, at least in the sand dune environment of the Simpson Desert. However, invertebrates are also included frequently in the diet and leaf, stem or root tissue from green plants is consumed to a lesser extent, supporting the trophic classification of this species as an omnivore rather than a strict granivore [15]. We suggest that omnivory generally should be advantageous in environments, such as those of arid Australia, that are characterized by marked and highly unpredictable fluctuations in climate and, consequently, in the food resource base that is available to consumers [25]. In such environments, the ability of foragers to utilize whichever food groups are available should reduce their likelihood of starvation compared with foragers that specialize in one food group, such as seeds. Our direct observations of foraging in *P. hermannsburgensis*, which are the first to be reported for any Australian desert rodents, provide further insights and intriguing questions about how animals find their food and the factors that affect the selection of food items. For example, animals spent only 0.99% of their time while active (69 of 6960 min) eating, with observations suggesting that they usually remain close to, or under, cover and that vigilance takes up a considerable portion of each animal’s time budget. Animals also appeared to dig only superficially for food items and clearly ignored seeds that were readily available on the soil surface before going on to select other, apparently similar, seeds. These observations suggest that the decisions made by *P. hermannsburgensis* about when and where to forage and what to eat are shaped by suites of factors that may be intrinsic (e.g., level of hunger) and/or extrinsic (e.g., risk of predation), and provide insight into avenues of research that will further uncover the foraging motivations of this species. 

## Figures and Tables

**Table 1:** Frequency of items of 3 main food types observed to be eaten by sandy inland mice *Pseudomys hermannsburgensis* during observations at night in the Simpson Desert. Percentage values for each time period are shown in parentheses.

Food Type	Spring	Summer	Autumn	Winter	Boom	Bust
Seed	14 (74)	12 (71)	21 (81)	18 (90)	27 (79)	38 (79)
Invertebrate	3 (16)	5 (29)	2 (8)	1 (5)	5 (15)	6 (13)
Green plant	2 (10)	0 (0)	3 (11)	1 (5)	2 (6)	4 (8)

**Table 2:** Frequency of items of 3 main food types in the stomachs (*n* = 176) of sandy inland mice *Pseudomys hermannsburgensis* collected in the Simpson Desert. Percentage values for each time period are shown in parentheses.

Food Type	Spring	Summer	Autumn	Winter	Boom	Bust
Seed	49 (49)	18 (55)	33 (44)	62 (52)	65 (58)	97 (45)
Invertebrate	36 (36)	13 (39)	31 (41)	45 (38)	33 (29)	92 (43)
Green plant	14 (14)	2 (6)	11 (15)	12 (10)	14 (13)	25 (12)

## Data Availability

Data are available upon reasonable request from the senior author.
